# Supplementary material for: Hybrid Models and Biological Model Reduction with PyDSTool
Source: PLoS Comput Biol. 2012 Aug 9;8(8):e1002628. doi: 10.1371/journal.pcbi.1002628 (PMC3415397; doi:10.1371/journal.pcbi.1002628)
Supplement: Text S4 — Complete source code for the PyDSTool package (version 0.88.120504). Includes API documentation and help files linking to web pages. This file is identical to the current public release on Sourceforge.net. (ZIP) [file pcbi.1002628.s004.zip › PyDSTool/html/PyDSTool.Toolbox.model_primitives-pysrc.html]

xml version="1.0" encoding="ascii"?


PyDSTool.Toolbox.model\_primitives


| Home | Trees | Indices | Help | PyDSTool |
| --- | --- | --- | --- | --- |

Package PyDSTool ::
Package Toolbox ::
Module model\_primitives

[hide private]

[frames] | no frames]

# Source Code for Module PyDSTool.Toolbox.model\_primitives

```
 1  """Library of common model equation primitives. 
 2   
 3  Contains functions, expressions, etc. 
 4  """ 
 5   
 6  from numpy import inf 
 7  from PyDSTool.MProject import ModelLibrary 
 8  import PyDSTool.ModelSpec 
 9  from PyDSTool.Symbolic import Fun 
10   
11  __all__ = ['threshold', 'linear_decay'] 
12   
13  # ------------------------------------------------------------------------- 
14   
15  threshold = ModelLibrary('threshold', 
16                          spectype = Fun, 
17                          pars={'k': [-inf,inf]}, 
18                          indepdomain={'x': [-inf,inf]}, 
19                          depdomain=[0,1], 
20                          description="""Smooth R -> [0,1] functions of 
21                          one parameter that represent thresholds""") 
22  tanh_spec = Fun('1+tanh(x/k)', ['x'], 'tanh_thresh') 
23  logistic_spec = Fun('1/(1+exp(-k*x))', ['x'], 'logistic_thresh') 
24  alg_spec = Fun('0.5*(1+x/(sqrt(k+x*x)))', ['x'], 'algebraic_thresh') 
25  # not yet supported 
26  #heav_spec = Fun('if(x>=0,1,0)', ['x'], 'heav_thresh') 
27  threshold.add_spec([tanh_spec, logistic_spec, alg_spec]) 
28   
29   
30  linear_decay = ModelLibrary('linear_decay', 
31                              spectype = Fun, 
32                              pars={'tau': [0,inf], 'x_inf': [-inf,inf]}, 
33                              indepdomain={'x': [-inf,inf]}, 
34                              depdomain=[-inf,inf], 
35                              description="""Linear decay given by two 
36                              parameters""") 
37  linear_decay.add_spec(Fun('(x_inf-x)/tau', ['x'], 'linear')) 
38
```

  


| Home | Trees | Indices | Help | PyDSTool |
| --- | --- | --- | --- | --- |

|  |  |
| --- | --- |
| Generated by Epydoc 3.0.1 on Fri May 4 15:24:22 2012 | http://epydoc.sourceforge.net |
